# Saccadic Eye Movement Characteristics in Adult Niemann-Pick Type C Disease: Relationships with Disease Severity and Brain Structural Measures

**DOI:** 10.1371/journal.pone.0050947

**Published:** 2012-11-30

**Authors:** Larry A. Abel, Elizabeth A. Bowman, Dennis Velakoulis, Michael C. Fahey, Patricia Desmond, Matthew D. Macfarlane, Jeffrey Chee Leong Looi, Christopher L. Adamson, Mark Walterfang

**Affiliations:** 1 Melbourne Neuropsychiatry Centre, University of Melbourne, Melbourne, Australia; 2 Neuropsychiatry Unit, Royal Melbourne Hospital, Melbourne, Australia; 3 Research Centre for the Neurosciences of Ageing, Academic Unit of Psychological Medicine, Australian National University Medical School, Canberra, Australia; 4 Department of Optometry and Vision Sciences, University of Melbourne, Melbourne, Australia; 5 Department of Paediatrics, Monash University, Melbourne, Australia; 6 Department of Radiology, Royal Melbourne Hospital, Melbourne, Australia; 7 Department of Radiology, University of Melbourne, Melbourne, Australia; 8 Murdoch Childrens Research Institute, Parkville, Australia; 9 Melbourne Brain Centre, Royal Melbourne Hospital, Department of Medicine, University of Melbourne, Melbourne, Australia; Barrow Neurological Institute, United States of America

## Abstract

Niemann-Pick Type C disease (NPC) is a rare genetic disorder of lipid metabolism. A parameter related to horizontal saccadic peak velocity was one of the primary outcome measures in the clinical trial assessing miglustat as a treatment for NPC. Neuropathology is widespread in NPC, however, and could be expected to affect other saccadic parameters. We compared horizontal saccadic velocity, latency, gain, antisaccade error percentage and self-paced saccade generation in 9 adult NPC patients to data from 10 age-matched controls. These saccadic measures were correlated with appropriate MRI-derived brain structural measures (e.g., dorsolateral prefrontal cortex, frontal eye fields, supplemental eye fields, parietal eye fields, pons, midbrain and cerebellar vermis) and with measures of disease severity and duration. The best discriminators between groups were reflexive saccade gain and the two volitional saccade measures. Gain was also the strongest correlate with disease severity and duration. Most of the saccadic measures showed strongly significant correlations with neurophysiologically appropriate brain regions. While our patient sample is small, the apparent specificity of these relationships suggests that as new diagnostic methods and treatments become available for NPC, a broader range of saccadic measures may be useful tools for the assessment of disease progression and treatment efficacy.

## Introduction

Niemann-Pick Type C (NPC) disease is a rare autosomal recessive disorder of lipid metabolism arising from mutations in the genes encoding for NPC1 and NPC2, lysosomal proteins that participate in intracellular sterol trafficking [1], [2]. Impairment in NPC1/2 function results in intracellular accumulation of cholesterol and gangliosides. In addition to visceral storage of glycosphingolipids, NPC particularly affects the central nervous system (CNS), resulting in ataxia, dystonia and cognitive impairment in all age groups. Childhood patients commonly also present with cataplexy and epilepsy, and adult patients frequently present with psychiatric illness such as psychosis in addition to developing a subcortical dementia.

One of the most reliably present neurological signs in adults is a supranuclear gaze palsy, affecting first vertical (particularly downwards) and later horizontal eye movements [1], [3]–[5]. As treatments for NPC are trialled, the need for reliable outcome measures becomes more important. Because saccadic eye movements can be readily quantified and their neural substrates are well understood, they are attractive as potential indicators of treatment efficacy. Ideally the more severely affected vertical eye movements would be examined, but because these may be nearly absent by the time diagnosis is established, horizontal saccades are generally assessed. Measures of horizontal saccadic function in NPC have focussed on a parameter related to the asymptotic peak saccadic velocity. This parameter is derived by dividing a saccade’s amplitude by its peak velocity and plotting the resulting duration against its amplitude, then fitting a linear regression line to the result and calculating the slope of that line. This slope is termed α [6]. It has been used to assess the efficacy of miglustat in the treatment of NPC [7], [8].

**Table 1:** Demographic and clinical characteristics of the NPC patient group.

Demographic Variables			Illness Variables			Biochemical Variables		Cognitive Variables	Clinical Ocular MotorFindings			
**Patient**	**Age (yrs)**	**Gender** **(M/F)**	**AOO** **(yrs)**	**DON** **(yrs)**	**Illness Scale**	**Filipin Staining %**	**Cholesterol Esterification** **(pmol/h/mg)**	**NUCOG Score** **(/100)**	**Vertical saccade impairment**	**Horizontal saccade impairment**	**Smooth pursuit impairment**	**VOR impairment**
**1**	49	M	47	2	5	20	NA	93.5	+	–	–	–
**2**	33	F	29	4	9	25	1.5	49	++	–	–	–
**3**	32	F	26	6	7	60	5.3	82.5	++	–	–	–
**4**	43	F	41	2	6	15	2.3	89	+	–	–	–
**5**	20	M	19	1	5	90	2	79	+	–	–	–
**6**	32	M	25	7	11	70	2.9	49	+++	+	–	–
**7**	31	M	19	12	15	95	0.3	51	+++	+	+	–
**8**	18	M	14	4	7	70	1.4	79	+	–	+	–
**9**	34	F	31	2	5	NA	NA	94	+	–	–	–

AOO = age of onset; DON = duration of neurological symptoms; NUCOG = Neuropsychiatry Unit Cognitive Assessment Tool; VOR = vestibulo-ocular reflex. + − detectable but no functional impairment; ++ − mild functional impairment; +++ − moderate to severe functional impairment.

**Figure 1:**
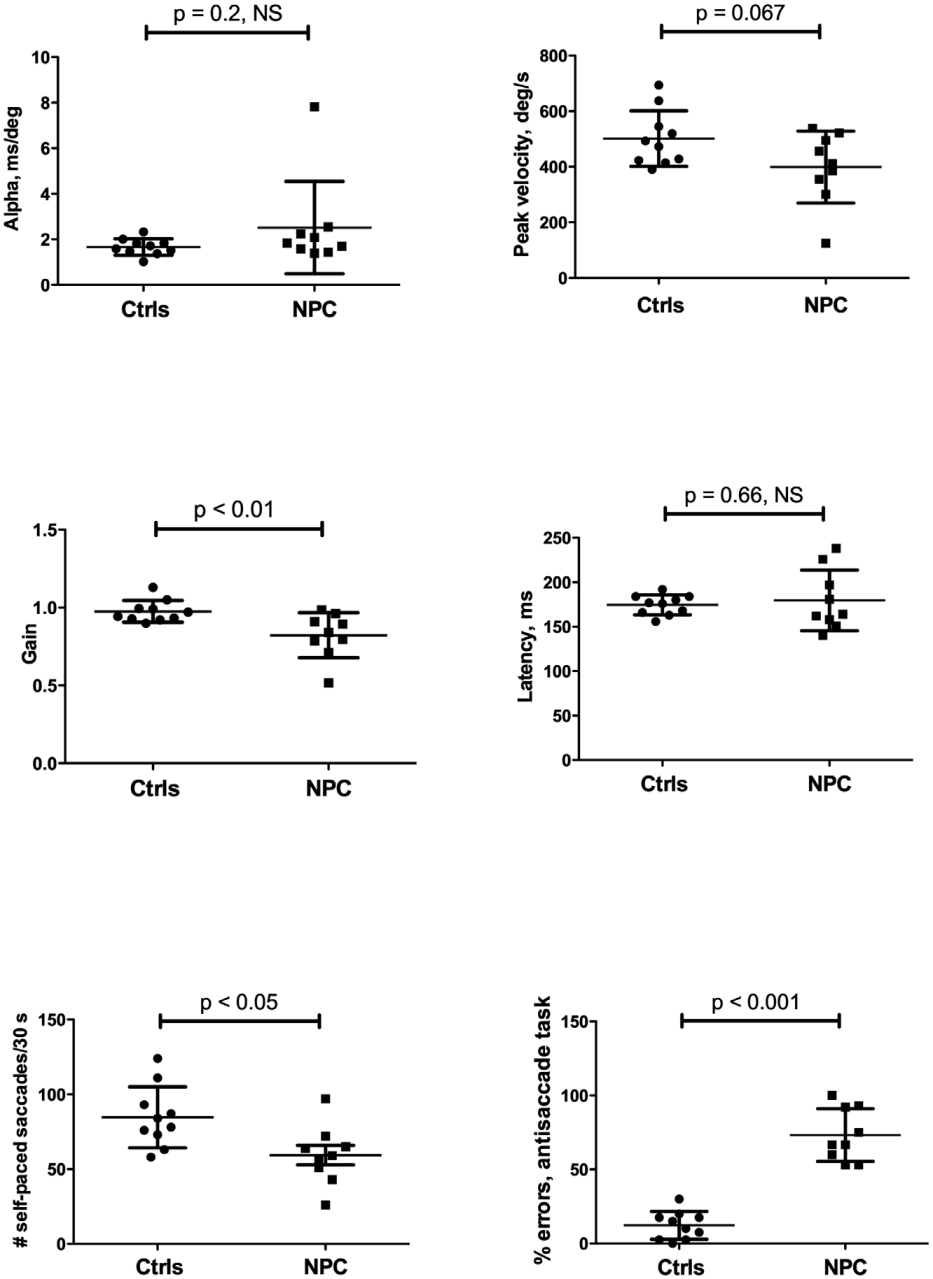
Comparisons of saccadic parameters between NPC patients and controls (error bars +/−1 S.D.) for a) horizontal saccade α, b) horizontal asymptotic peak velocity Vmax, c) gain, d) latency, e) self-paced saccade rate over 30 s and f) percentage of antisaccade errors.

Saccadic peak velocity largely depends upon the integrity of brainstem burst neurons, located in the paramedian pontine reticular formation (PPRF) for horizontal saccades and rostral interstitial nucleus of the medial longitudinal fasciculus (riMLF) for vertical saccades [4]. While these regions are appreciably affected by NPC, pathology is more widespread across the CNS and may include frontal structures which have been implicated in the psychiatric symptoms of NPC [9] and which are also involved in other aspects of ocular motor control [10], [11]. These functions include the frontally-mediated initiation of volitional saccades and the inhibition of unwanted reflexive saccades. Disturbances of volitional saccade control have been found to be impaired in conditions as diverse as attention deficit-hyperactivity disorder, Alzheimer’s disease, schizophrenia, Tourette’s syndrome and traumatic brain injury [12]–[18]. Volitionally generated saccades rely more than reflexive ones on the integrity of frontal cortical regions which contain the frontal eye fields (FEF), supplementary eye fields (SEF) and dorso-lateral prefrontal cortex (DLPFC) [10], [19]. Antisaccade error suppression is also highly dependent upon the integrity of the DLPFC, [10], [11], located in the middle and superior frontal gyri [11], [20]. More posteriorly, lesions of the parietal eye field (PEF) have also been reported to cause hypometria in reflexive saccades [20]. Furthermore, lesion studies have strongly implicated the cerebellar vermis as a key region for maintaining reflexive saccadic gain [21]–[23].

Because we have no *a priori* reason to assume that a treatment being evaluated would equally benefit all brain regions affected by NPC, we felt it potentially useful to expand the range of saccadic parameters evaluated and previously reported on these in a small group of adult NPC patients [24]. Our study group has since expanded to nine patients and we present here the results of their saccadic evaluation in comparison with a group of ten age-matched control subjects. We also examine the relationships between these saccade parameters and assessments of both clinical illness and of MRI-based measures of brainstem, cerebellar and cortical regions which are the putative neural substrates of the saccadic parameters, thus extending both the number of patients studied and the range of assessments made beyond the earlier paper [24].

**Table 2:** Correlations of saccadic parameters with illness duration and severity measures.

DON (yrs)	Vmax	α	Reflexive latency	Gain	Antisaccade error %	Self-paced saccade rate (/30 s)
Pearson r	−.804	0.838	−0.085	−0.913	0.77	−0.586
p	0.009*	0.005*	0.828	0.001*	0.015*	0.097
**Illness scale**						
Pearson r	−0.797	0.826	0.055	−0.909	0.669	−0.674
p	0.01*	0.006*	0.889	0.001*	0.049	0.046
NUCOG Score						
Pearson r	0.505	−0.493	−0.259	0.723	−0.371	0.672
p	0.165	0.177	0.501	0.028	0.326	0.047

In this and subsequent tables an asterisk (*) indicates correlations which remained significant after Holm-Bonferroni correction for multiple comparisons.

## Methods

### Subjects

Nine patients (5 male, 4 female, mean age 32.4±9.71 years) recruited from the Royal Melbourne Hospital were assessed between 2007 and 2011. Some findings in these patients have been reported elsewhere [24], [25] All patients provided written informed consent and the study was approved by the Melbourne Health human research and ethics committee (HREC 2005.198). Diagnosis was confirmed with biochemical analysis of cultured fibroblasts, using cholesterol esterification rate and percentage of cells staining abnormally for perinuclear cholesterol. Duration of illness was measured by years of neurological symptoms, and illness severity rated on the NPC-specific Iturriaga rating scale [26]. Cognition was assessed using the NUCOG, a multidimensional bedside cognitive assessment tool that measures attention, memory, executive function, language, and visuospatial function [27]. Patient characteristics are shown in Table 1. Ten normal subjects (2 men, mean age 28.9±4.43 years), matched for age but not for sex with the patients (mean ages did not significantly differ with Student’s t-test) and free of any neurologic or psychiatric condition or medication known to affect eye movements, completed the same ocular motor tests as the patients.

### Ocular Motor Assessment

Testing procedures in the NPC patient group have been previously described [24]. Briefly, horizontal eye position was recorded with a Microguide 1000 infrared limbus eye tracker and digitised along with a target position signal at 1000 Hz. Stimuli were green 5 mm LEDs on an arc 1.6 m in front of the participant. Only data from the better-recorded eye were analysed, given conjugate gaze in NPC. For calibration, the target stepped from 0 to 20 degrees left and right every 5 seconds. For reflexive saccades, 60 target steps between 5 and 30 degrees amplitude were randomly presented. For antisaccades, 40 targets at ±5 and 10 degrees were presented randomly, with participants instructed to look away from the stimulus. For self-paced saccades, targets at ±10 degrees were illuminated and the participant asked to look between them as rapidly as possible for 30 seconds. Data were analysed interactively under Matlab with a program that identified saccade onset and offset using both velocity and acceleration thresholds of 30 deg/sec and 8,000 deg/sec^2^; reflexive saccade gain and latency were calculated, as was the peak velocity of each saccade. This was used to calculate asymptotic peak velocity Vmax, obtained from the nonlinear relationship.

V = Vmax(1– e^-amp/C^). We also calculated α, the slope of the peak duration versus amplitude plot [6]. The gain of each saccade (the ratio of saccade amplitude to stimulus amplitude) was also calculated. Antisaccade errors were tallied and the percentage of erroneous responses calculated. Finally, the number of self-paced saccades generated in 30 seconds was counted by direct inspection. The mean values obtained for the NPC patients were compared to those of the control subjects using unpaired t-tests.

**Table 3:** Correlations of peak velocity-related parameters with brainstem areas.

Vmax	Midbrain area	Pontine area	PMR
Pearson r	0.759	−0.221	−0.886
p	0.018*	0.569	0.002*
α			
Pearson r	−0.573	0.214	0.758
p	0.107	0.580	0.018

**Table 4:** Correlations of reflexive latency with brainstem areas.

Reflexive latency	Midbrain area	Pontine area	PMR
Pearson r	−0.153	−0.855	−0.161
p	0.694	0.003*	0.679

**Table 5:** Correlations of reflexive gain with parietal areas containing the PEF.

Reflexive gain	Left superior parietal	Left postcentral	Right superior parietal	Right postcentral
Pearson r	0.828	0.563	0.860	0.439
p	0.006*	0.114	0.003*	0.237

### Neuroimaging

All subjects were scanned on a 1.5T GE Signa MRI machine at the Royal Melbourne Hospital. A volumetric spoiled gradient recalled echo (SPGR) structural sequence generated 124 contiguous, 1.5 mm coronal slices with TE/TR 3.3/14.3 ms; flip angle, 30deg; matrix size, 256×256; field of view, 24×24 cm; voxel dimensions, 0.938×0.938 mm. We examined three chief brain regions involved in saccadic function: the brainstem, cerebellum and frontal/parietal cortex. Brainstem structure was examined using the mid-sagittal area of the pons, midbrain, and the pontine:midbrain ratio, a measure we have shown to index disease state in adult NPC [25]. Briefly, pontine and mibrain areas were manually outlined on the mid-sagittal slice, and the respective areas calculated using the image analysis software ANALYZE 10.0 (Mayo BIR, Rochester, NY, USA) and from the ratio of these mid-sagittal areas the pontine:midbrain ratio was calculated (The area delineation is detailed in [25]). Cerebellar regions were calculated on patients using the SUIT (Spatially Unbiased Infratentorial Template) toolbox V2.3.4 for SPM8 [28], which segments infratentorial structures, and then undertakes a nonlinear deformation to a high-resolution template of the cerebellum from 20 healthy individuals and then defines cerebellar lobuli and vermis according to a probabilistic cerebellar atlas. Cortical regions were parcellated using Freesurfer 5.0 [29] which uses a combination of affine transformation, intensity normalization [25] and non-linear registration, and mapping of the cortical surface representation to a standard spherical co-ordinate system, correcting for topology and using surface warping to align anatomically homologous points before parcellating into cortical regions.

### Statistical Analysis

The calculated saccade parameters were compared between patient and control groups using Student’s t-test. Correlations were carried out between the saccadic parameters and illness severity and years of neurological illness scores, as well as with the measures of brain region areas obtained as described previously, using Pearson’s r. Brain regions were selected on the basis of known involvement of the selected areas with the saccadic parameters under examination. Correction for multiple comparisons was undertaken using the Holm-Bonferroni correction, which controls the family-wise error rate (FWER) at a given level of alpha to minimise type I errors whilst performing multiple Bonferroni tests at each intersection [30], whilst also minimising the type II errors inherent in a simple Bonferroni correction.

**Table 6:** Correlations of self-paced saccade rate with frontal areas containing DLPFC, FEF and SEF.

Self-paced saccaderate (/30 s)	Left rostral mid-frontal	Left precentral	Left paracentral	Right rostralmid-frontal	Right precentral	Right paracentral
Pearson r	0.802	0.634	0.092	0.886	0.564	0.148
p	0.009*	0.067	0.814	0.001*	0.114	0.704

## Results

### Comparison of Patient and Control Groups

Horizontal saccade α, the parameter chosen for the miglustat clinical trial [7], did not differ significantly between patient and controls groups (Fig. 1a); neither did Vmax, the parameter to which it is approximately inversely proportional (Fig. 1b), although its difference approached significance. Reflexive saccade latencies were nearly identical between the two groups (Fig. 1c), suggesting that our adult NPC patients demonstrate no impairment in saccade initiation. In contrast, the gain of patients’ reflexive saccades was significantly reduced (Fig. 1d), reflecting impairment in the correct calculation of the required amplitude, resulting in hypometric saccades. Both volitional saccade parameters were significantly impaired in the NPC patients–as a group they could generate significantly fewer self-paced saccades in 30 sec (Fig. 1e) and were particularly poor at suppressing unwanted reflexive saccades during the antisaccade task (Fig. 1f). This was not due to task non-comprehension, as all patients were able to produce at least a few correct antisaccades during practice trials. Variability in each measure was considerable, however.

### Correlation of Saccadic and Clinical/radiological Measures

When the saccadic parameters Vmax, α, gain and antisaccade error percentage were correlated with illness duration and severity, significant correlations were found between illness severity and Vmax, α and gain (Table 2), while illness duration correlated with antisaccade error rate in addition to the aforementioned parameters. Cognition as measured by the NUCOG correlated with gain and self-paced saccades, but not after significance levels were corrected.

As reported previously [25], Vmax significantly correlated with midbrain area and the pontine/midbrain ratio (PMR) (Table 3). Reflexive saccade latency was correlated only with pontine area (Table 4). Correlations between saccadic gain and lobuli VI through×of the posterior vermis were not significant, although vermis lesions have been reported to reduce gain [21], [22]. Correlations between gain and the postcentral and superior parietal gyri, that contain the PEF [19] (Table 5) were highly significant.

Volitional saccade control was also examined with respect to relevant anatomical regions [10], [11], [20]. Correlations of percentage of antisaccade error trials with these regions were low and not significant. Self-paced saccade rate was also correlated with frontal cortical regions that contain the FEF, SEF and DLPFC [10], [20]; the correlations between left and right mid-frontal gyri, said to contain DLPFC, were significant (Table 6).

## Discussion

This paper is the first to make group comparisons on a wide range of saccade parameters between a group of adult NPC patients and a group of age-matched controls. Unlike previous studies [3]–[5], [7], [8], [31] the measures examined drew on structures ranging from the brainstem to prefrontal cortex. Relationships were also examined between these measures and indicators of disease severity and duration. It is noteworthy that in the present study, the parameter used as a measure of treatment response in the miglustat clinical trial [7]–horizontal saccade α [6]–neither differed significantly between groups nor correlated significantly with any of the brainstem anatomical areas. This does not invalidate its utility as a measure of treatment response, however, as it was significantly correlated with both illness severity and years of neurological symptoms, consistent with the clinical observations that as the disease progresses, saccadic dysfunction spreads from the vertical to the horizontal plane. The related asymptotic peak velocity parameter Vmax showed similar relationships, though the comparison between the NPC and control groups approached significance. Its strong negative correlation with the pons/midbrain ratio (PMR) appears paradoxical but, as has been reported elsewhere [25], PMR in this instance may be a particularly reliable marker for overall disease progression. Support for this comes from the fact that this previous study also reported that self-paced saccade rate, a parameter with no plausible relationship to either midbrain or pontine areas, was also correlated inversely with PMR. The reliability of the PMR as a neuroimaging measure enhances its utility for correlative studies such as the present.

In contrast to the lack of likely anatomical specificity between the velocity measures and brainstem anatomical regions, reflexive saccade latency was strongly negatively correlated with pontine area (Table 2). If a reduction in pontine mid-sagittal area corresponded to a loss of the omnipause neurons which inhibit burst neurons until a saccade is wanted, we might expect a positive correlation, with reduced area leading to reduced inhibition of excitatory burst neurons. Perhaps a loss of pontine volume reflects the loss of these burst neurons themselves, with a concomitant impairment of those remaining, thus increasing their response time. More precise imaging of the relevant brainstem regions than is currently available, able to resolve individual nuclei, would be necessary to resolve this question.

While neither velocity measure discriminated significantly between NPC and control groups, other measures did. Reflexive saccade gain was significantly lower in patients than in controls and it also showed the strongest negative correlations (r>−0.9) with both illness severity and years of neurological symptoms, suggesting it is a powerful illness marker. Although cerebellar ataxia is a common symptom of NPC [1], [2], the failure of gain to correlate with measures of cerebellar vermis size suggest that hypometric saccades in NPC may be driven less by cerebellar pathology than from parietal cortical involvement.

Two measures reflect functions strongly related to the integrity of frontal cortex and the projections from it: the ability to inhibit unwanted reflexive saccades in the antisaccade task and the ability to rapidly initiate volitional saccades between two fixed targets. Both of these differed between groups, especially antisaccade errors, for which the distributions of the two groups failed to overlap. Only the relationship between antisaccade error percentage and years of neurological illness remained significant after correction for multiple comparisons and no correlations were found to be significant between this measure and measures of the frontal regions containing the DLPFC. This may reflect a ceiling effect, as a number of subjects found the antisaccade task nearly impossible and none of the patients’ performance on the task approached normal limits. Generating self-paced saccades was expected to draw upon the substrates of both FEF and possibly the supplementary eye field (SEF), given the predictive nature of the movements required; however, the only significant correlations either before or after correction were with regions containing DLPFC. While not generally associated with saccade initiation, it has been reported that DLPFC lesions impair the ability to predict the motion of predictable saccade stimuli [11]. Perhaps the resemblance of responses on the predictive task to the repetitive saccades between two fixed locations is sufficient for this region to be of importance in the self-paced task.

### Limitations

The most obvious limitation of this study is the small sample size. This is unavoidable given the rarity of NPC as a disease, and the fact that adult patients may account for only 20–30% of the patient population as a whole. In spite of the small sample, a number of the findings appear quite robust, even after correction is made for multiple comparisons. The small sample also precludes stratification by sex or mutation. Sex-matching controls was not possible but only one study has reported a sex difference in relevant saccadic parameters, finding females to have shorter latencies and better accuracy (values were not given) [32]; however, this was only detectable with their sample of 245 subjects. Our neuroimaging methodology largely relies on semi-automated rather than fully manual tracing methodologies, which may increase the risk of mis-registration and boundary estimation errors; this would be expected to increase variance in measures and reduce between-group differences or strength of correlations, suggesting that our current significant findings hold. There is also the possibility that the various results may all be indicators of disease severity; however, the specificity of some of the correlations suggests that for at least some of the parameters we are seeing indications of pathology in specific brain regions.

### Conclusion

As new treatments become available for neurodegenerative disorders such as NPC, the ability to evaluate the stabilisation or reversal of disease activity at specific locations in the brain may be important. This will be particularly true if faster and more readily available diagnostic techniques become available beyond skin fibroblast culture [33]. One potentially very important advance is the detection of oxysterol markers in plasma of affected individuals [34]; a simpler and more widely available assay may allow for the earlier diagnosis of individuals with NPC, who currently may go many years before being properly identified [9]. This could render those parameters currently too impaired to be treatment indicators at the point of diagnosis, such as vertical saccade velocity and gain or identification of antisaccade errors, useful biomarkers. Horizontal saccade α has already been used as an assessment of treatment response in randomized controlled trial of miglustat in NPC [7]; future studies, particularly those able to follow patients over time, could readily add parameters such as gain, along with the more frontally mediated measures such as self-paced saccades and antisaccade errors, enhancing their ability to obtain more localised information about both disease progression and treatment response. Thus, while the pattern of saccadic abnormalities is not unique to NPC, overlapping with conditions such as progressive supranuclear palsy, frontotemporal lobar dementia and corticobasal degeneration [35], the range of deficits offers the potential to monitor treatment responses across multiple brain regions.
